# Hydrogen Peroxide and Polyamines Act as Double Edged Swords in Plant Abiotic Stress Responses

**DOI:** 10.3389/fpls.2016.01343

**Published:** 2016-09-12

**Authors:** Kamala Gupta, Atreyee Sengupta, Mayukh Chakraborty, Bhaskar Gupta

**Affiliations:** ^1^Department of Biological Sciences, Presidency UniversityKolkata, India; ^2^Department of Botany, Government General Degree College, Affiliated to University of BurdwanSingur, India; ^3^Department of Zoology, Government General Degree College, Affiliated to University of BurdwanSingur, India

**Keywords:** polyamines, hydrogen peroxide (H_2_O_2_), reactive oxygen species (ROS), nitric oxide (NO), abscisic acid (ABA), abiotic stress tolerance

## Abstract

The specific genetic changes through which plants adapt to the multitude of environmental stresses are possible because of the molecular regulations in the system. These intricate regulatory mechanisms once unveiled will surely raise interesting questions. Polyamines and hydrogen peroxide have been suggested to be important signaling molecules during biotic and abiotic stresses. Hydrogen peroxide plays a versatile role from orchestrating physiological processes to stress response. It helps to achieve acclimatization and tolerance to stress by coordinating intra-cellular and systemic signaling systems. Polyamines, on the other hand, are low molecular weight polycationic aliphatic amines, which have been implicated in various stress responses. It is quite interesting to note that both hydrogen peroxide and polyamines have a fine line of inter-relation between them since the catabolic pathways of the latter releases hydrogen peroxide. In this review we have tried to illustrate the roles and their multifaceted functions of these two important signaling molecules based on current literature. This review also highlights the fact that over accumulation of hydrogen peroxide and polyamines can be detrimental for plant cells leading to toxicity and pre-mature cell death.

## Introduction

Life and stress go hand in hand for all living organisms but in case of plants, being sedentary organisms, stress has to be dealt with in a special way. Plants are subjected to constant environment changes forcing them to fine tune their metabolic processes in order to maintain a steady state balance of the energy production and consumption. A dedicated-signaling network influencing the three main metabolic processes—photosynthesis, respiration, and photorespiration—help in overcoming the imbalance, thereby maintaining growth, and productivity. The main fallout of metabolic imbalance is oxidative stress caused due to the excess production of reactive oxygen species (ROS). Therefore, in order to maintain normal growth and development the plants orchestrate a myriad of stress responsive metabolites like proline and polyamines, along with several antioxidative enzymes, that help to detoxify the ROS. Recent studies have also revealed the capability of ROS to act as signaling molecules in activating defense responses (reviewed by Gill and Tuteja, 2010; Gupta et al., 2013a; Pál et al., 2015; Saha et al., 2015). Thus, ROS are considered nowadays as not only toxic byproducts of aerobic metabolism with strictly controlled cellular levels, but they also function as signaling agents regulating many biological processes and producing pleiotropic effects (Gadjev et al., 2008; Mittler et al., 2011).

Polyamines—putrescine, spermidine, and spermine—which are present in multitude of living organisms are a group of low molecular weight polycations with diverse physiological and developmental functions essential for events such as senescence and stress responses (Roy et al., 2005; Gupta et al., 2014; Nahar et al., 2016). Counterbalancing cellular levels of ROS, in order to maintain a healthy environment for the cells to thrive, is one of the major roles played by polyamines (Miller et al., 2010; Saha et al., 2015). Hydrogen peroxide (H_2_O_2_) is one of the key ROS molecules produced in living cells from various internal sources. Particularly in plants, the major processes that lead to the production of H_2_O_2_ involve photorespiration or C_2_ cycle, which includes three different organelles—chloroplast, mitochondria, and peroxisome. Of these, mitochondrial and chloroplastidial electron transport chain and oxidation of fatty acids in the mitochondrial matrix play a major role in contributing to the H_2_O_2_ pool within the cell. In addition, pathogenic infections might also induce an oxidative stress leading to oxidative burst. In fact both the production and scavenging of H_2_O_2_ act in synchrony to tide out plants during stress conditions (Miller et al., 2010).

The paradox of H_2_O_2_ physiology is indeed an interesting one—on one hand at lower concentrations it initiates various intra cellular signaling activities while at higher concentrations it is malevolent for the cellular metabolites (Gechev and Hille, 2005; Bhattacharjee, 2012). ROS levels when too high might lead to metabolic dysfunctioning of plant cells and at the same time induce nucleic acid, protein, and lipid damages (Anjum et al., 2015). To combat oxidative stress, plants produce metabolites and molecules like polyamines and H_2_O_2_ (Mittler et al., 2011). Sub-cellular organelles like the mitochondria and chloroplasts are also key regulators in the sense that alterations in carbon metabolism during stress in these compartments also help in metabolic coordination to avoid excessive generation of ROS and oxidative damage (Takahashi and Murata, 2008; Baxter et al., 2014).

The focus of this review is to decipher the roles of the two important players—H_2_O_2_ and polyamine—either antagonistic or agonistic or both and to try to elucidate a relationship between them, which eventually modulate the signaling cascades that are initiated in response to abiotic stress.

## ROS and polyamines—key players in abiotic stress response

**Figure 1:**
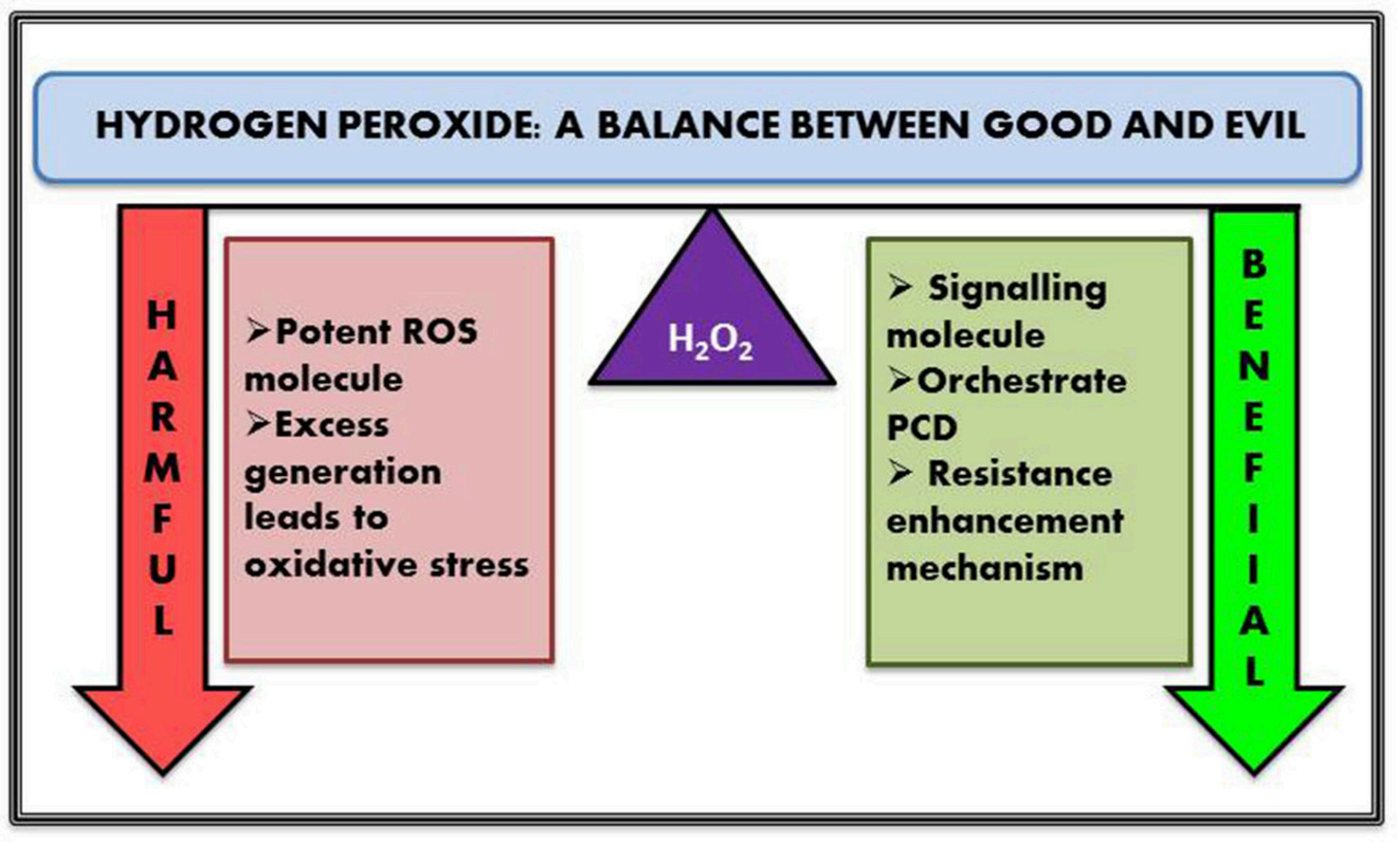
**Dual role of intercellular hydrogen peroxides**.

In this modern era of food security, the ever-increasing level of population demands a robust scientific approach for proper harvest and increase of food crops. From drought to salt and metal toxicity to temperature—stress conditions are omnipresent and have to be dealt with properly without creating any adverse effect on the essential metabolome. The crop productivity of the entire world in this era of food security remains a matter of great concern. It has been observed for a long time that most damage to crop plants in fields occur when two or more stresses are prevailing (Mittler, 2006). Most recent studies indicate that the plant's responses to two or more factors are unique and differ from the response to one factor only. For example, subjecting the plants to only drought stress leads to high content of proline, but subjecting the same species to drought combined with high temperature leads to high content of sucrose and other sugars, but not proline. Hence, Mittler (2006), studying all prevailing abiotic factor has suggested treating this situation as a new stress condition that he called “stress combination.” Several studies have established the role of ROS as a key signaling molecule in initiating defense mechanism in response to environmental stresses and pathogen infections by modulating pathways involved in different developmental processes and programmed cell death (PCD) (Mittler et al., 2011; Baxter et al., 2014). Different abiotic stress factors such as drought and salinity have been found to augment the production of ROS and lead to ROS-associated injury (Miao et al., 2006; Abbasi et al., 2007; Zhu et al., 2007; Giraud et al., 2008). ROS scavenging properties have also been identified by the increased cellular accumulation of sugar alcohols such as mannitol, sorbitol, and inositol. Transgenic tobacco with increased mannitol concentration confers protection to the cell from oxidative damages by increasing its scavenging capacity. Moreover, mannitol accumulations do not show any deleterious effect on plants, thus proving that sugar does not show any malicious feedback (Bolouri-Moghaddam et al., 2010). Intensity of light has also remained a potent stress for plants and the triggering of common pathways has been illustrated in a number of studies. The main effect of high light intensity is the damage to the chloroplasts and the antennae complex (Davletova et al., 2005; Moller et al., 2007; Triantaphylidès et al., 2008). The maximal efficiency of PSII and the accumulated ROS, share a linear relationship between each other. Thermal damage to photosystems leads to less absorption of photon. In severe thermal stress conditions, the photons absorbed by PSI and PSII are considered as surplus electrons, and serve as the source of ROS (Moller et al., 2007). High atmospheric CO_2_ levels have been found to stimulate photosynthesis in C3 plants because of decreased photorespiration, which is widely accepted as a major source of H_2_O_2_ (Miller et al., 2008). Therefore, low CO_2_ level activates ROS generation leading to the activation of abiotic and biotic stress responses (Munné-Bosch et al., 2013). H_2_O_2_ mediates the expression of a number of genes—H_2_O_2_ induced transcripts have been found to play an important role and encoded proteins with functions such as metabolism, cellular energy production, protein destination and transport, cellular organization and biogenesis, cell rescue of defense, and transcription (Desikan et al., 2001; Neill et al., 2002) (Figure 1). Activation of signaling molecules such as Ca^2+^, salicylic acid (SA), abscisic acid (ABA), jasmonic acid (JA), ethylene, nitric oxide (NO) have been reported to be directly or indirectly linked with H_2_O_2_ signaling and vice versa. More studies have provided evidence that H_2_O_2_ itself is a key signal molecule mediating a series of molecular stress responses, being a part of the signaling cascade (Desikan et al., 2004), and activating many other important signal molecules (Ca^2+^, SA, ABA, JA, ethylene, NO) of plants (Schieber and Chandel, 2014; Vaahtera et al., 2014). These signal molecules function together and play a complex role in signal transduction of resistance responses, and growth and development in plant. H_2_O_2_ as a signaling molecule has drawn attention in the last decade or so and ample evidence has been found which supports these assumptions (Dat et al., 2000; Baxter et al., 2014; Saha et al., 2015). Apart from its role as master player in plant stress response regulator it was also reported as the most basic key ingredient in regulating several physical and physiological aspects of plant growth and development such as cell cycle, stomatal movement, photosynthesis, photorespiration, and senescence (Bright et al., 2006; Mittler et al., 2011).

Exogenous application of molecules like polyamines has remained an important genre of studying ways to ameliorate stress in plants (Roy et al., 2005; Farooq et al., 2009; Gupta K. et al., 2012; Gupta S. et al., 2012; Sengupta et al., 2016). Abiotic stress causes drastic changes in the pathways involved in the metabolism of N_2_ and polyamine. The exact role of these polycationic molecules had remained undefined for many years. With the use of model systems like *Arabidopsis thaliana*, there has been a global approach in deciphering the role of the polyamines and unveiling its metabolic pathway (Ferrando et al., 2004). According to recent studies, the maintenance of proper equilibrium of its catabolic and anabolic pathways along with the modulations of H_2_O_2_ level during these processes indeed help plants to tide over stress and adapt properly to the surrounding environment. Recent studies indicate that the redox gradient across the plasma membrane is a key sensor of global climatic change and a crucial regulator of redox signaling (Munné-Bosch et al., 2013). The impact of this global climate change on agriculture will be enormous and it is essential for our survival to note various aspects of H_2_O_2_ function and crosslinks with regulatory molecules like polyamines.

## Polyamines—anabolism, catabolism, and conjugation

**Figure 2:**
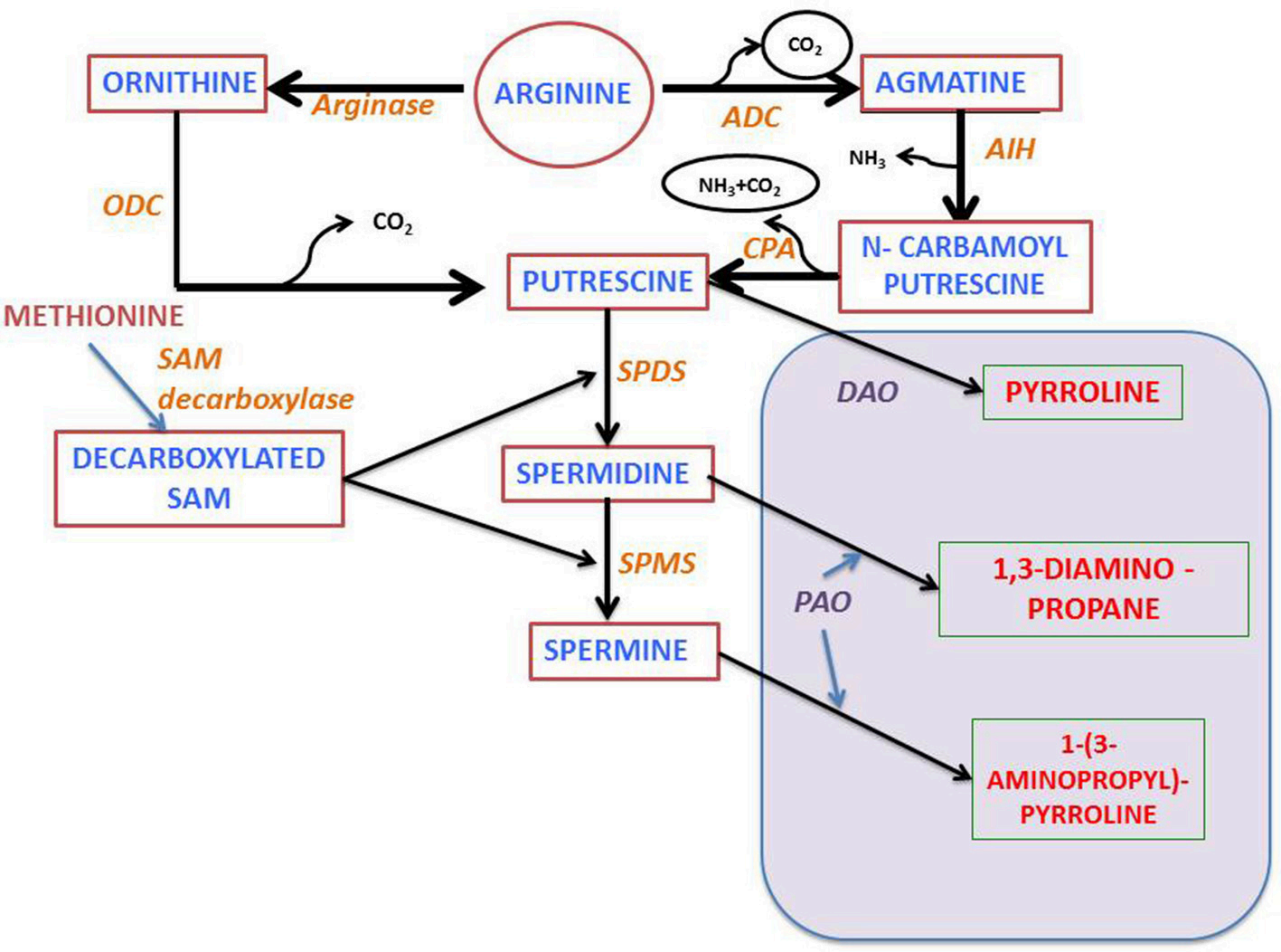
**Polyamine metabolism: PA, Polyamine; ODC and ADC, Ornithine or arginine decarboxylase; AIH, agmatine iminohydrolase; CPA, N-carbamoylputrescine amidohydrolase; DAO, diamine oxidase; SPDS, spermidine synthase; SPMS, spermine synthase; SAM, S-adenosylmethionine; PAO, polyamine oxidase (see text for further details)**.

Polyamine biosynthesis in plants progress through the decarboxylation step(s) of ornithine or arginine (Figure 2). In the presence of enzymes, namely either ornithine or arginine decarboxylases (ODC or ADC), the diamine putrescine is formed. The ADC pathway, which yields putrescine, consists of three sequential enzymatic steps, beginning from agmatine iminohydrolase (AIH) and ending at N-carbamoyl putrescine amidohydrolase (CPA). Sequential addition of aminopropyl groups to putrescine and spermidine leads to synthesis of higher molecular weight polyamines by the activity of spermidine synthase and spermine synthase. SAM decarboxylase helps in generating the amino-propyl groups (Figure 2). Analysis and characterization of genes encoding these enzymes in *Arabidopsis* has shown that in this plant there is only ADC activity and the ODC activity is not detectable (Hanfrey et al., 2001), whereby indicating that putrescine is produced exclusively through the ADC pathway. Moreover, it has been found that in *Arabidopsis* there are two genes encoding ADC (*ADC1* and *ADC2*), a single gene, each for AIH and CPA (Janowitz et al., 2003; Piotrowski et al., 2003) and at least four for SAM decarboxylase (*SAMDC1, SAMDC2, SAMDC3*, and *SAMDC4*) (Urano et al., 2004). Also, it has been further observed that there are two genes for spermidine synthase (*SPERMIDINES1* and *SPERMIDINES2*).

Both anabolic and catabolic pathways regulate the levels of polyamine whose intracellular levels depend not only on their biosynthesis but also on catabolic and conjugation reactions. Two main enzymes namely amine oxidases, in particular diamine oxidases (DAO) and polyamine oxidases (PAO), catalyze the deamination of polyamines. DAOs display high affinity for diamines, while PAOs oxidize secondary amine groups from spermidine and spermine. The *A. thaliana* genome contains 12 genes with sequence homology to *DAO*s from other species, and 5 *PAO* genes (Moschou et al., 2012). Polyamines not only form base conjugates but also conjugate with other macromolecules like proteins and hydrocinnamic acid (reviewed by Hussain et al., 2011; Gupta et al., 2013a). Enzymes which are involved in the conjugation reactions are transglutaminase and putrescine hydroxycinnamoyl transferase respectively (Bagni and Tassoni, 2001). In *Arabidopsis*, one gene encoding the enzyme transglutaminase has been characterized (*AtPng1*) (Della Mea et al., 2004). However, till date putrescine hydroxycinnamoyl transferase encoding gene has not yet been identified. Involvement of secondary metabolites in plant abiotic stress response has been widely studied using *A. thaliana* as a model plant (Ferrando et al., 2004). Arabidopsis whole genome sequencing (Arabidopsis Genome Initiative 2000) enabled researchers to identify all the genes that are involved in polyamine anabolic and catabolic pathways. Genome-wide expression profiling of genes involved in abiotic stress responses provided a global view on plant defense mechanism in light of polyamine metabolism (e.g., using AtGenExpress; Kilian et al., 2007). Apart from genome level analysis, transcriptomic approach is another well-established method used for recognizing the underlying interrelationship among the abiotic stress induced transcripts along with their *cis*-regulatory elements (Kilian et al., 2007). A similar kind of work was carried out by Ma and Bohnert (2007), where they correlated the presence of specific *cis*-regulatory motifs with the expression profile of several stress induced genes. They observed that these stress regulatory motifs are profoundly involved in modulating the functions and expression of genes that show differential responses when exposed to abiotic stress. Gong et al. (2016) suggest that SlSAMS1 (S-adenosylmethionine synthetase 1) function as a stress mediatory element enhancing alkali stress tolerance through polyamine and H_2_O_2_ cross-linked networks in tomato callus. This provide us with a new insight in plant's alkali stress tolerance mechanism.

Several genes involved in polyamine biosynthetic pathways have so far been already reported to be upregulated when exposed to one or combination of one or more abiotic stresses. Putrescine is the first polyamine that is accumulated in cells on exposure to abiotic stress. Interestingly, increase in putrescine concentration leads to the induction of enzymes that are responsible for the conversion of putrescine to spermidine and spermine, through auto-regulation process. Concentration of putrescine, spermidine, and spermine varies greatly within the cell mostly because of the simultaneous degradation pathway which occurs within the cell, whereby generating H_2_O_2_. This H_2_O_2_, generated as a result of polyamine catabolic pathway, cause oxidative stress on one hand while on the other it plays an essential role in lignification of cell wall, thus protecting plant from adverse effect of stress. Modulating the level of endogenous polyamine by regulating biosynthetic genes is an important procedure for studying the role of polyamine metabolism in stress alleviation (Alcazar et al., 2006).

## H_2_O_2_ production and cellular distribution—a necessary evil

**Figure 3:**
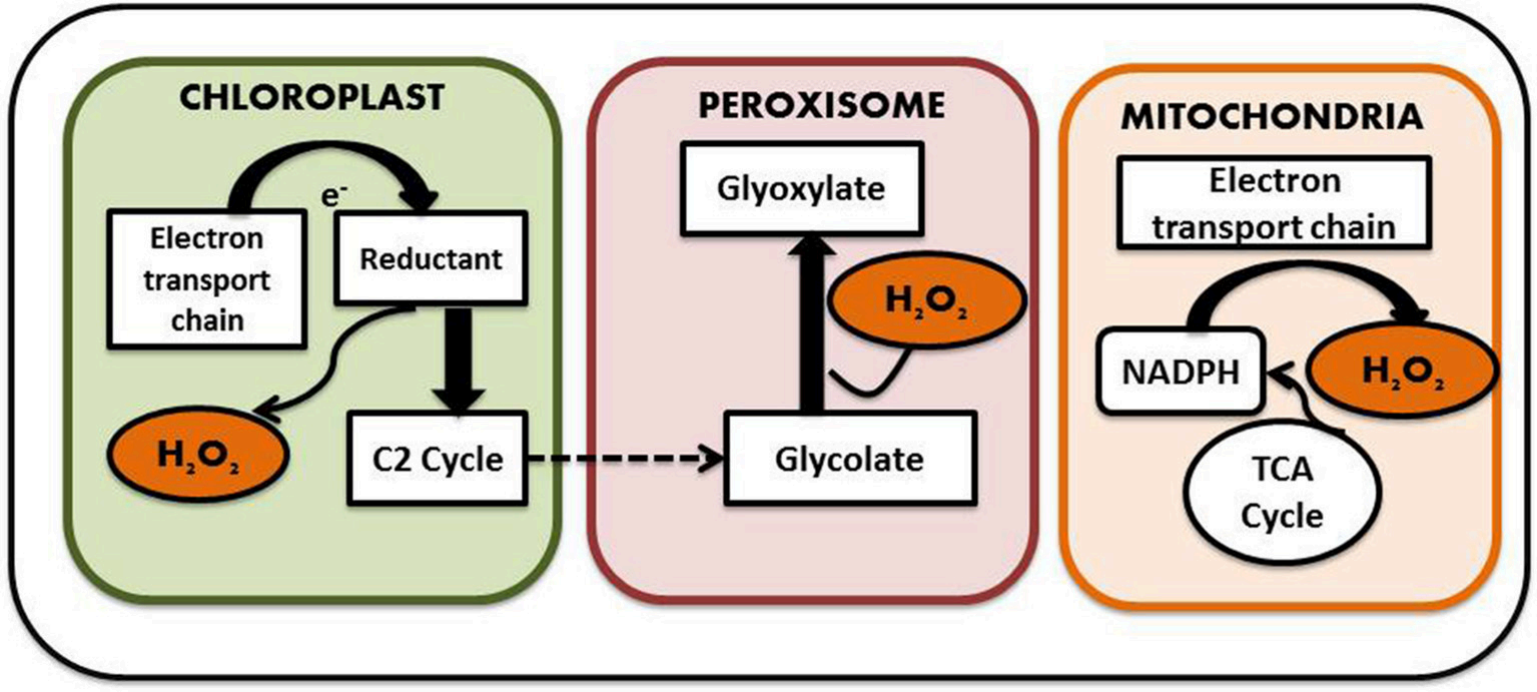
**Subcellular localization of H_**2**_O_**2**_ (see text for details)**.

Generation of ROS is considered as an indispensible outcome of aerobic metabolism, which comes along with its share of goodness and evilness. A plethora of ROS species have been identified in plants including H_2_O_2_, superoxide anion (${\text{O}}_{2}^{-}$), hydroxyl radicals (OH), singlet oxygen (^1^O_2_), and nitric oxide (NO) and surprisingly most of them are interconvertible. For instance superoxide molecules on reduction yields H_2_O_2_, which on further reduction liberates water and hydroxyl radical. Cellular oxidation reactions involving these molecules have just the reverse sequence. Studies have shown that only 0.1% of the total oxygen consumed by the plants is diverted for the production of ROS (Bhattacharjee, 2005). ROS is considered as a necessary evil as it functions in several developmental and adaptive responses in both animal and plant cells while its excess generation leads to severe oxidative damage. So it is necessary to maintain a balance between the beneficial and deleterious effects demonstrated by ROS for proper cellular function. Among the different intracellular ROS species, H_2_O_2_ is considered as one of the most prevalent one. In contrast to other ROS mentioned above, it has a relatively long half-life and can be produced in all cell compartments. Moreover, as it is highly diffusible, it can easily pass membranes. The endogenous H_2_O_2_ content of plant cells is usually much higher than that found in animals and bacteria; plant cells happily survive with H_2_O_2_ levels that would kill animal cells. This tolerance is linked to the presence in plant cells of highly efficient antioxidant systems described in detail later on Costa et al. (2010). It is generated by a number of ways in plants. Malfunctioning chloroplast and mitochondrial electron transport chain serves as one of the major source of H_2_O_2_ generation in plant cells. The process is carried out by membrane bound NADPH Oxidases, also known as respiratory burst oxidase homologs (rbohs), which are regulated by a unique class of Rho-like proteins called ROPs (Rho-related GTPases from plants) as well as by cell wall-associated peroxidases (Agrawal et al., 2003). NADPH Oxidases initially reduce molecular O_2_ to superoxide molecule with simultaneous oxidation of NADH to FAD. Superoxide molecule thus produced is converted into H_2_O_2_ by the action of another enzyme known as superoxide dismutase. Some form of peroxidases (type III POX), in addition to their role in oxidation of phenolics required for cell wall loosening and stiffening, can generate H_2_O_2_ coupled with the oxidation of NADH (Andronis et al., 2014). In addition, there are several flavin containing limited-substrate oxidases like peroxisomal glycolate oxidase, glyoxisomal xanthine oxidase and urate oxidase, which directly produce H_2_O_2_ (Delrio et al., 1992). A sulfite oxidase localized in the peroxisome has also been identified to have a role in production of H_2_O_2_ (Hänsch et al., 2006). Apart from these, oxidases such as DAO and PAO, which are involved in the polyamine degradation pathways, also serve as source to the H_2_O_2_ pool. Not only stress but also normal physiological conditions can lead to ROS production as part of various metabolic pathways (Ahmad, 2014). For example, oxygen molecule, which is produced as the byproduct of mitochondrial electron transport chain, is sometimes reduced to superoxides that are in turn dismutated to form H_2_O_2_. Mitochondrial electron transport chain comprises of four distinct enzyme complexes. They are NADH dehydrogenase (Complex I), succinate dehydrogenase (Complex II), ubiquinone-cytochrome C oxidoreductase (Complex III), and cytochrome oxidase (Complex IV). Electron transfer occurs involving either complex I, III, and IV or complex II, III, and IV, leading to the generation of molecular O_2_. The molecular O_2_ generated can be reduced to superoxide and the reduction is catalyzed by ubiquinone of complex III, which serves as one of the major site for ROS—such as superoxide and H_2_O_2_ generation. Complex III bears two ubiquinone binding sites, one is located near the inner surface of the inner mitochondrial membrane while the other one is on the outer surface, which indicates the presence of ROS on both luminal and matrix side of this membrane. Not only the mitochondrial electron transport chain, but the chloroplast electron transport chain also transfers electron from photosystem II to NADP thereby yielding reduced NADPH which is used during the Calvin cycle for reduction of CO_2_. This also serves as a potent site for superoxide anion and H_2_O_2_ generation. Other subcellular organelles that actively participate in H_2_O_2_ production are peroxisomes and glyoxisomes (present only in plants) which carries out several reactions including beta oxidation of fatty acids and light dependent oxidation of glycolate to glyoxylate by glycolate oxidase (Foyer and Noctor, 2005) (Figure 3). Thus, it is understandable that generation of H_2_O_2_ is an irrevocable process irrespective of its consequences.

H_2_O_2_ plays a versatile role in plants—at mild concentration it acts as a signal molecule and is involved in the alleviation of various abiotic and biotic stresses (Jaspers and Kangasjarvi, 2010; Mittler et al., 2011; Dietz et al., 2016 and its references). At the same time higher cellular concentration of H_2_O_2_ orchestrates unwarranted PCD and leaf senescence in plants (Dat et al., 2000; Gadjev et al., 2008). H_2_O_2_ also takes part in plant cell wall reinforcement (lignification, cross-linking of cell wall structural proteins), phytoalexin production and resistance enhancement against different forms of stresses (Gill and Tuteja, 2010; Ahmad, 2014). In case of biotic stress, H_2_O_2_ production in plants might trigger killing of the pathogen either directly or by prompting defense genes to limit infection by the microbe. Hypersensitive responses are the master players behind establishment of plant immunity against disease causing pathogens. It is another well-known approach that leads to PCD in plant thus inhibiting pathogen invasion. H_2_O_2_ is the key signaling molecule that actively participates in mediating hypersensitive responses by triggering localized host cell death. It has been also reported to play a crucial role in regulating hormone mediated signaling and vice versa (Pei et al., 2000). A classic example of hormone-mediated response is stomatal closure, where presence of H_2_O_2_ is perceived by histidine kinase receptor ETR1, which further transduces the signal and ensures the closing of stomata (Bright et al., 2006). Efficiency of any signaling molecule lies in the fact that they are rapidly produced and removed immediately once it has accomplished its role, and H_2_O_2_ fits the bill perfectly. They are produced rapidly by various cellular processes and quenched promptly. In addition, it can easily react with different biomolecules such as membrane lipid, carbohydrate, protein and DNA and can effortlessly diffuse through aquaporins, thus generating further molecular responses. Apart from its role in hypersensitive reaction and PCD, H_2_O_2_ induces the expression of glutathione-S-transferase and glutathione peroxidase encoding genes. H_2_O_2_ treated *E. coli* cells exhibited 140 upregulated mRNA transcripts in DNA microarray experiments—thus revealing the role of H_2_O_2_ in signal transduction (Li et al., 2001). A similar kind of study was carried out by Desikan et al. (2001) in arabidopsis where about 175 non-redundant EST's were reported those of which are modulated by H_2_O_2_. Generally signaling molecule activates its receptor by complimentary binding—however, in case of H_2_O_2_, signal transduction occurs via chemical reactions. Oxidation of cysteine residue of a receptor protein is considered as one of the major path for mediating intracellular signals (Paulsen and Carroll, 2010). There are also evidences that suggest their interaction with secondary messengers such as MAP kinase and their activation. These activated MAP kinase molecules in turn activate different transcription factors thus initiating intricate signaling cascades (Asai et al., 2000). It is interesting to note that H_2_O_2_ generated from different cellular organelles unveil a plethora of molecular responses. For example H_2_O_2_ produced from chloroplast was observed to be involved in early signaling responses such as activation of transcription factors and secondary messenger biosynthetic genes while H_2_O_2_ generated from peroxisomes are mostly involved in upregulating the genes involved in protein repair pathway (Sewelam et al., 2014).

H_2_O_2_ seems to share a unique inter-relationship with NO and Ca^2+^. H_2_O_2_ and NO together have been reported to play an essential role in plant developmental and physiological processes such as growth of pollen tube, growth and development of root, closing of stomata, delayed senescence etc. (Serrano et al., 2012; Huang et al., 2015; Iakimova and Woltering, 2015; Shi et al., 2015). Not only developmental processes, they together play an active role in abiotic stress alleviation as well. They protect drought stressed leaf mesophyll tissue from damage and also increase the activity of myo-inositol phosphate synthase in drought stressed plants (Liao et al., 2010). They have been reported to increase low temperature tolerance by mediating polyamine oxidation in *Medicago* (Guo et al., 2014). There are several other evidences that confirm the role of both H_2_O_2_ and NO in remitting abiotic stress. However, the mechanism behind their interaction is still not very clear. Most probably H_2_O_2_ functions as a cofactor in endogenous NO synthesis. This view has been endorsed by the findings of Lin et al. (2012) and Shi et al. (2015). NO on the other hand can regulate stomatal closure in H_2_O_2_ mutant and in wild type plant treated with H_2_O_2_ scavengers and inhibitors. Thus, it can be clearly said that both of their production pathways are inter-related and can regulate production of each other. Other than with NO, H_2_O_2_ has been found to share a distinctive bond with Ca^+2^ as well. Endogenous Ca^2+^ influx increase in arabidopsis root epidermis on application of exogenous H_2_O_2_. This increase in Ca^2+^ influxes in the root plasma membrane of arabidopsis seedling leads to root elongation. Ca^2+^ influx is probably mediated by H_2_O_2_ dependent activation of Anexin 1 protein, which is a Ca^2+^ transport protein, thus promoting its growth and development (Demidchik et al., 2007; Richards et al., 2014; Han et al., 2015). Another striking example that demonstrates the inter-relationship between H_2_O_2_ and Ca^2+^ is H_2_O_2_ dependent adventitious root formation, which involves Ca^2+^ signaling. In fact the removal of intracellular Ca^2+^ prevents formation of adventitious root. Pei et al. (2000) established a clear association between H_2_O_2_, Ca^2+^ and stomatal closure using patch clamp techniques, thus connecting ABA signaling cascades with stomatal closure mediated by H_2_O_2_ and Ca^2+^ channels. Thus, all available evidences point toward an intricate crosstalk between H_2_O_2_ and Ca^2+^.

So considering all the roles played by H_2_O_2_—either good or bad—be it as stress alleviator, secondary messenger, chemo-selective signaling molecule or stress inducer, it surely deserves the tag “Jack of many trades.”

## Scavengers employed to limit the oxidative damage

Generation of ROS and aerobic life goes hand in hand, and these two phenomena are inseparable. So to deal with the situation—living organisms have evolved several ROS scavenging mechanisms—such as administration of enzymatic and non-enzymatic antioxidants that confer protection against oxidative stress. Among the various ROS species that are present within a cell, H_2_O_2_ is most stable with a half-life of 1 ms while the half-life of others are much shorter, i.e., about 2–4 μs (Bhattacharjee, 2005). In general H_2_O_2_ is considered as a weak oxidizing agent. A number of investigations have revealed that ROS, especially H_2_O_2_, plays an important role related to the signal transduction involved in plant adaptation to the changing environment (Pei et al., 2000; Neill et al., 2002; Moon et al., 2003; Terzi et al., 2014; Reczek and Chandel, 2015). Although the presence of H_2_O_2_ sensor protein still remain unelucidated, it is presumed that H_2_O_2_ is directly perceived by the redox transcription factors, showing redox sensitivity, which in turn activates the downstream signaling cascades. It also participates in several physiological pathways of plant and activates defense responses to various stresses. H_2_O_2_ is beginning to be accepted as a second messenger due to some features that are exclusively present in all secondary messenger molecules. It is mobile, small and can diffuse in and out of the cell freely thereby relaying signals between different cellular compartments, thus modulating different biological processes (Neill et al., 2002). However, excess accumulation of H_2_O_2_ adversely affects the plants by leading to oxidative stress. Therefore, presence of efficient H_2_O_2_—scavenging mechanisms is a compulsory criterion for all aerobic organisms. Antioxidative enzymes, and certain non-enzymatic antioxidants (Miller et al., 2008; Sung et al., 2011; Saha et al., 2015) work in tandem and maintain a sinuous but delicate balance to detoxify H_2_O_2_. Among the wide array of antioxidative enzymes that function in scavenging ROS species, Catalase (CAT), Ascorbate peroxidase (APX), and Peroxidases (POX) require special mention as they act directly upon the H_2_O_2_ molecules, thus reducing it to water. SOD carries out the dismutation reaction by reducing ${\text{O}}_{2}^{-}$ molecule to H_2_O_2_ whereas CAT, APX, GPX are involved in converting H_2_O_2_ to water thus ensuring its removal. Based on the presence of metal ion, SOD can be classified into three different types—(i) Mn-SOD, which is located in the mitochondrial matrix and peroxisome, (ii) Cu/Zn-SOD, which is present in large quantities in the chloroplast and cytosol, and (iii) Fe-SOD, which is localized only in chloroplastids of higher plants. All of them function together when the plant is exposed to unfavorable conditions thus protecting the cells from probable damage. H_2_O_2_ which is generated as a result of superoxide dismutation is removed by enzymes such as CAT, five different isoforms of APX and different isozymes of GPX. These biological catalysts are localized in all the organelles where H_2_O_2_ generating enzymes are present such as peroxisome, glyoxisome, cytosol, mitochondria etc. CAT is the major enzyme involved in the quenching of H_2_O_2_ with highest enzyme turnover rate. Studies have revealed that about six million H_2_O_2_ molecules can be converted to H_2_O and O_2_ per minute by one molecule of CAT, thereby making CAT one of the major enzymes involved in H_2_O_2_ detoxification. Apart from CAT another enzyme that plays an equally important role is APX, which catalyses the first step in AsA-GSH cycle and works in coordination with ascorbic acid and glutathione (Asada, 2006; Fan and Huang, 2012; Begara-Morales et al., 2013; Jahan and Anis, 2014). Apart from the enzymatic antioxidants some non-enzymatic antioxidants such as α-tocopherol, ascorbic acid (AsA), glutathione etc. also play a vital role in sustaining stable redox state by removing excess ROS from different cellular compartment, thus detoxifying the cell. AsA that is synthesized in mitochondria, is uniformly distributed throughout the plant and serve as a substrate for APX enzyme, which reduces H_2_O_2_ to water, yielding mono-dehydroascorbate (MDA) in ascorbate-glutathione cycle (AsA-GSH cycle) (Gapper and Dolan, 2006). It helps regulating α-tocopherol level in cells and is also involved in biosynthesis of zeaxanthine pigment which protects the plant from photo-oxidation or photo-bleaching (Foyer and Noctor, 2005; Munné-Bosch, 2007), thus pursuing an important role in oxidative stress tolerance. Several studies have revealed that application of exogenous AsA diminishes the harmful effect of oxidative stress (Hossain et al., 2011). Glutathione, another non-protein thiol, and a non-enzymatic antioxidant, also plays an essential role in H_2_O_2_ scavenging (comprehensive review by Noctor et al., 2012). Conversion of reduced glutathione (GSH) to oxidized glutathione (GSSG) and its ratio is considered as a marker for determining redox balance within the cell. Functionally it is almost similar to AsA but in addition to that it also plays an eminent role in heavy metal chelating. Therefore, both glutathione and ascorbic acid are now contemplated as the chief ingredients of redox signaling. Moreover, another group of biomolecules that should be mentioned in this context are polyamines. As mentioned earlier, polyamines are positively charged molecules which protect the cell from oxidative damage both directly and indirectly. Directly it functions as an antioxidant itself thus scavenging free radicals and indirectly it has been reported to regulate the level of enzymatic and non-enzymatic antioxidants within the cell (Groppa and Benavides, 2008). Thus, increase in polyamine concentration is often correlated with increase in stress tolerance. However, on the other hand polyamine catabolism is one of the major contributors of H_2_O_2_ to the H_2_O_2_ pool. Endogenous polyamines, like H_2_O_2_, play a dual role by both adding and diminishing the concentration of H_2_O_2_ within the cell, thereby maintaining a thin line of balance between their molecular concentrations. Since the maintenance of appropriate H_2_O_2_ concentration within the cell is of utmost importance for carrying out normal physiological and developmental processes and to combat abiotic stress in plants, efficient ROS scavenging mechanism along with adequate polyamine concentration is of vital importance for its sustainable growth and survival.

## Role of polyamine in oxidative stress

**Figure 4:**
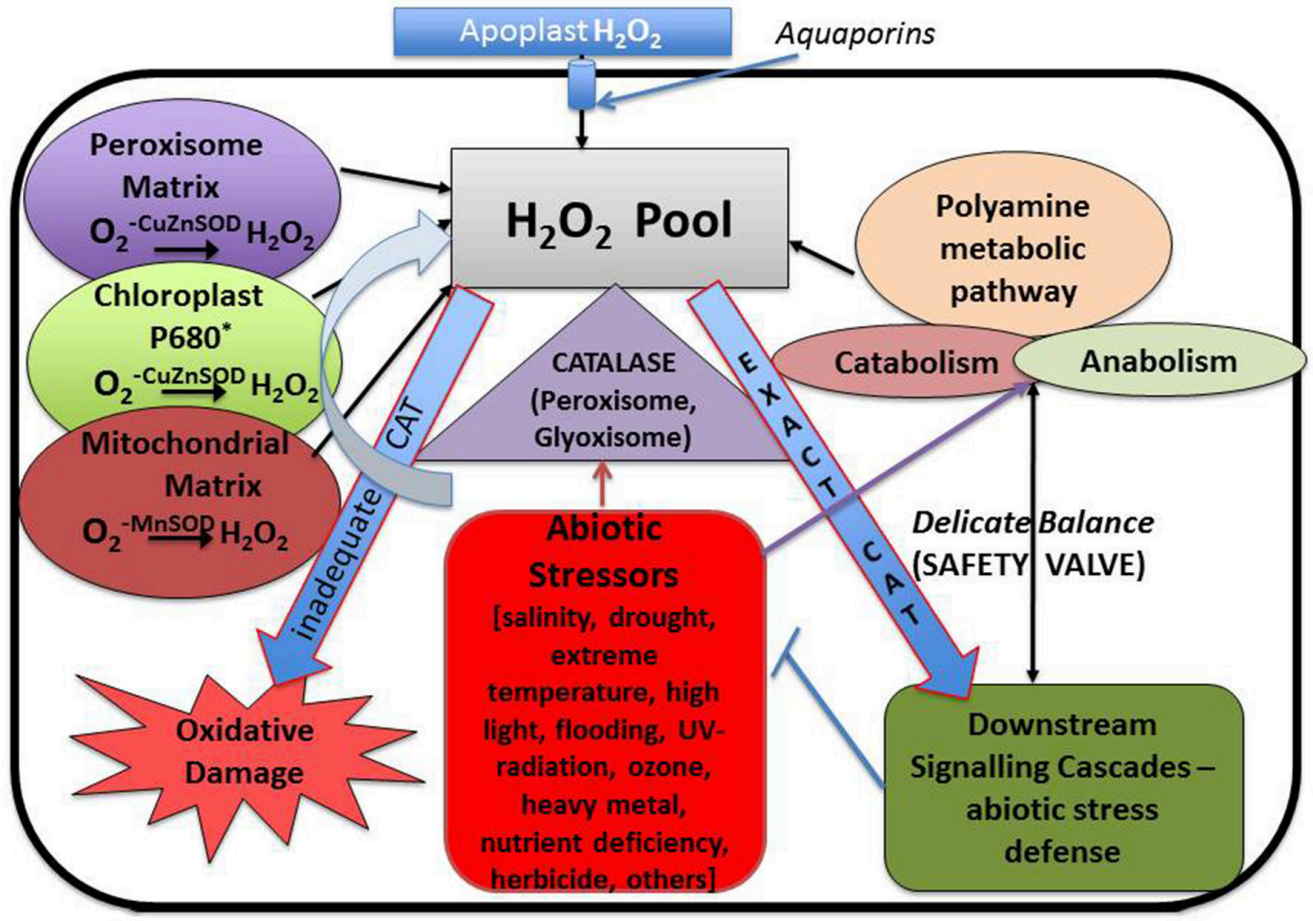
**Interrelationship between cellular hydrogen peroxide, polyamine metabolic pathway and different forms of abiotic stresses with special emphasis on the role played by “catalase” antioxidant enzyme**.

Plant stress biologists have marked oxidative stress as an after effect of each and every abiotic stress—be it heavy metal accumulation, excess salinity, high and low temperature or scarcity of water. Each of these abiotic stresses is associated with the generation of excess ROS, leading to oxidative damage to cellular components. Studies have revealed that application of exogenous polyamines alleviate abiotic stress, thereby conferring stress tolerance. Abiotic stress is known to impair cellular membranes through their interaction with the membrane structure or as a result of ROS-mediated peroxidation of membrane lipids (Anjum et al., 2015). The antioxidative effect of polyamines can be attributed to a combination of their anionic and cationic-binding properties in radical scavenging, inhibiting properties of lipid peroxidation, metal-catalyzed oxidative reaction, and production of H_2_O_2_ by DAO and PAO (Groppa and Benavides, 2008). Free and bound polyamines are reported to be modulated by UV-B radiation in different plant species (Mapelli et al., 2008) thereby protecting them against ozone damage and ozone-derived oxidative damage (Groppa and Benavides, 2008). H_2_O_2_ produced by polyamine catabolism may cause activation of antioxidative defense responses. Phenylpropanoid-polyamine conjugates are known to act as antioxidants against ROS and reactive nitrogen species in response to stress conditions (Yamasaki and Cohen, 2006). Shen et al. (2000) reported that spermidine may act as a cellular membrane protectant against chill-induced lipid peroxidation in cucumber through prevention of activation of superoxide-generating NADPH oxidase. As discussed in the earlier section, CAT enzyme plays an essential role in regulating the balance between excess and exact amount of cellular H_2_O_2._ Moreover, polyamines directly or indirectly modulate the level of CAT enzyme when exposed to abiotic stress (Figure 4), thus forming an intricate stress tolerance network. Sung et al. (2011) have demonstrated the role of polyamines in mitigating hypersalinity-induced oxidative stress in a marine green macroalgae (*Ulva fasciata*) by modulation of antioxidative enzyme (FeSOD, MnSOD, CAT, APX) gene expressions. Radhakrishnan and Lee (2013) observed that, in comparison to untreated plants, CAT activity decreases in polyethylene glycol (PEG) treated soya bean plants simulating drought environment. However, the enzyme activity transiently increases when the plants are treated with PEG along with polyamine spermine. A similar kind of experiment was carried out by Farooq et al. (2009), using rice plant as the experimental model. It was observed that exogenous treatment with spermine successfully ameliorated the effect of osmotic stress by accelerating the activity of CAT enzyme. In another study, acid rain fed bean plants showed a sudden burst in the H_2_O_2_ level which in turn decreased the CAT activity in the cell. This decrease in the CAT activity is probably due to the binding of H_2_O_2_ molecule to the enzyme active site, thus deactivating the enzyme (Sharma et al., 2012; Mittova et al., 2015). However, with longer exposure to acid rain, activity of CAT was found to increase, hence conferring stress tolerance. However, in bean plants pretreated with polyamines (spermine or spermidine), the rapid generation of H_2_O_2_ was checked. This may be due to the protective shield imparted by the polyamines on the membrane which stabilize it (Velikova et al., 2000). Verma and Mishra (2005) demonstrated that under conditions of high salt concentration *Brassica* plants show an increased level of H_2_O_2_ in plants untreated with polyamines, whereas in polyamine treated plants the level of H_2_O_2_ decreases considerably. It was also observed that CAT activity increases when exposed simultaneously to NaCl and polyamine, rather than when exposed to NaCl alone, thereby establishing the role of polyamines in protecting the plants from oxidative injury (Verma and Mishra, 2005). Exogenous application of polyamines reduced the H_2_O_2_ and malondialdehyde (MDA) content, and increased the antioxidant levels in drought and cold stressed 15-day-old chickpea plants (Nayyar and Chander, 2004). DNA oxidative degradation by OH^−^ was inhibited in the presence of spermine in *Mesembryanthemum crystallinum* (Kuznetsov and Shevyakova, 2007) illustrating the efficiency of polyamines as free radical scavengers. According to some research groups, polyamine conjugates show more efficient antioxidant activities compared to their parent compounds (Edreva et al., 2007; Hussain et al., 2011).

**Figure 5:**
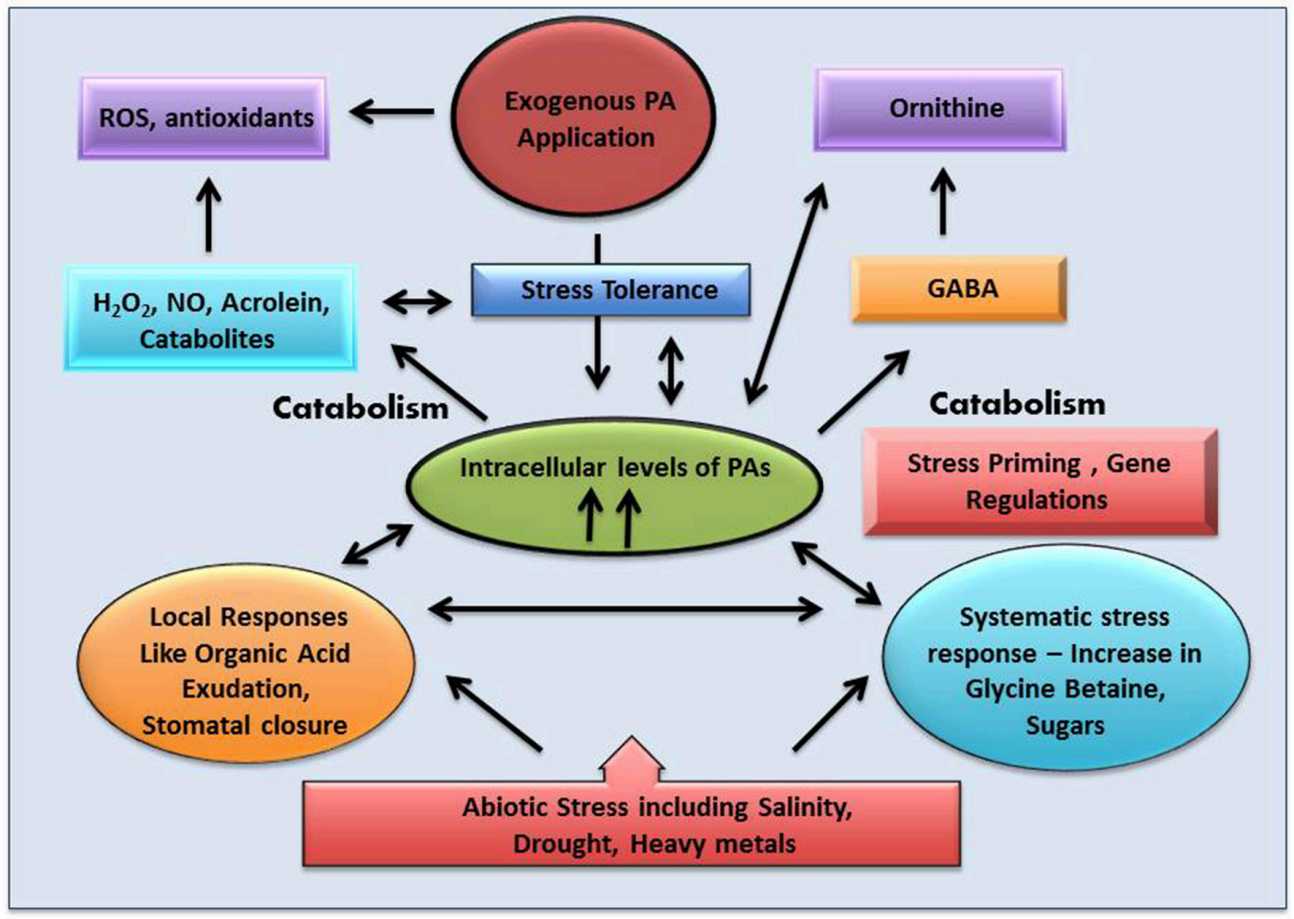
**An overview of the role of polyamine (PA) in plant abiotic stress tolerance**.

Under metal stress CAT shows a differential response. In some plants like *Glycine max, A. thaliana*, and *Capsicum annuum* CAT activity decreases, while in *Brassica juncea, Oryza sativa*, and *Triticum aestivum* its activity increases. Another very interesting observation was reported by Hsu and Kao (2007) where it was shown that pretreating a plant with H_2_O_2_ increases the CAT activity which in turn protects the plant from cadmium induced oxidative damage. A similar kind of trend was observed in salt treated *Cicer arietinum*. A completely opposite trend was noticed by Sharma and Dubey (2007) in drought treated rice seedling, where CAT activity decreases. This observation was congruent with the observation made by Pan et al. (2003) where CAT activity decreases in *Glycyrrhiza uralensis* seedlings when exposed to both salinity and drought stress. From the above observations it may be concluded that the increase or decrease in CAT activity primarily depends on the nature of sensitivity toward stress of a particular plant. In sensitive variety CAT level tends to increase. On the contrary, in stress tolerant variety the level of CAT activity decreases. Several reports demonstrate that polyamine plays an interesting role in modulating the CAT level thus regulating the H_2_O_2_ content of the cell. So it is easy to hypothesize an inter-relation between endogenous and exogenous polyamines, CAT enzymes and stress generated H_2_O_2_. Most probably, they function in a loop. Oxidative stress leads to generation of H_2_O_2_ which signals activation of CAT enzyme and endogenous polyamine—CAT functions in removal of H_2_O_2_ molecule and polyamines protect the membrane from oxidative damage thus conferring a protective shield. Application of exogenous polyamines strengthen the ROS removal procedure in varieties where CAT activity decreases in response to stress thus forming a perfect interrelated network of tolerance (Figure 5). Polyamines have been instrumental in reducing protein carbonylation and tyrosine nitration while subsequently increasing protein S-nitrosylation.

**Figure 6:**
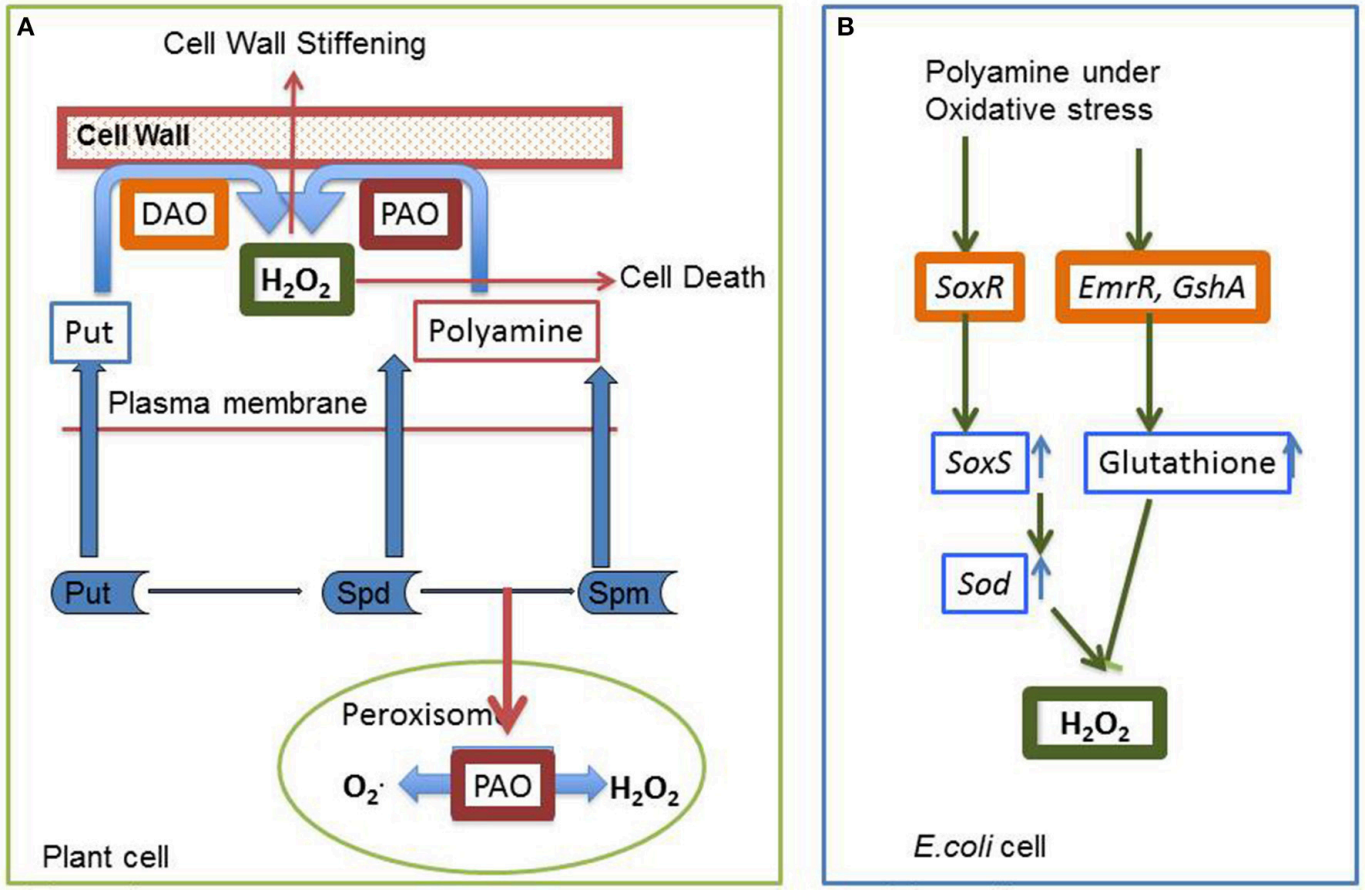
**Interaction of polyamine and H_**2**_O_**2**_ (A) In plant cells H_**2**_O_**2**_ is generated during the process of polyamine catabolism, that may be utilized as a signaling molecule, PCD or cell wall stiffening**. PAO in peroxisome maintains a delicate balance between O_2_∙ and H_2_O_2_ **(B)** In simple prokaryotic bacterial cell like in *E. coli*, polyamines have been reported to be involved in ameliorating oxidative stress through stimulation of two transcription factors SoxR and EmrR (see text for details).

Previous results have shown that plants employ polyamine catabolism derived H_2_O_2_ as a defensive contrivance against abiotic stress (Cona et al., 2006). Tanou et al. (2014) have reported increase in intracellular *DAO* and *PAO* activity in plants treated with excess salt (Figure 6A). Treatment with NaCl alone have shown to increase both O2^•−^ and H_2_O_2_ production, indicating existence of an oxidative stress situation. It was inferred that in the presence of salt, endogenous polyamines induce the generation of O2^•−^. However, exogenous polyamine treatment lowers O2^•−^ level, with significant difference being observed after spermine application. In addition, H_2_O_2_ content strongly increases in putrescine or spermidine-treated plants compared to those treated with salt alone. Exogenous polyamine application on salt treated plants shows an increase in endogenous polyamine level when compared to plants which are not treated with polyamines, thereby confirming the beneficial role of extracellular polyamine in mitigating salt stress (Shi et al., 2010). Polyamines have been reported to be taking part in inter-organ signals in plants. Moreover, it was observed that putrescine administration evoke spermidine accumulation in roots on exposure to salinity, whereas spermidine treatment enhances spermine production in leaves, illustrating the metabolic conversions of polyamines in the case of the entire plant. Likewise, the addition of spermine increases the endogenous spermidine and putrescine concentration in roots whereas spermidine application increases leaf putrescine concentration in salt treated citrus, thus depicting the possible conversions that might occur on exposure to a single polyamine under abiotic stress conditions (Tavladoraki et al., 2006; Moschou et al., 2008a,b). Studies have divulged that exogenous application of polyamines in salt treated roots stimulates polyamine biosynthetic genes in the leaves, asserting its universal systematic role (Kuznetsov et al., 2002). Further evidences came from the work of Tassoni et al. (2008), who demonstrated the advantageous effect of free spermidine on Arabidopsis flower under high salt concentration. Another extremely interesting phenomenon observed in *Vitis vinifera* revealed that stress exposure causes an immediate rise in the putrescine level; however, increase in spermine/spermidine level occur much later (Liu et al., 2011). Photochemical efficiency of PSII which is often hampered by stress was found to be enhanced by external application of putrescine (Zhang et al., 2009). Rice plants treated with spermidine for the first 7 days after germination followed by their continuous exposure to salinity till maturity shows a better grain yield and increased ion content in comparison to those that are not treated with spermidine for the initial 7 days (Saleethong et al., 2013). Thus, it is evident that ROS scavenging mechanisms in co-ordination with polyamines play important role during plant abiotic stress adaptation.

## H_2_O_2_ and polyamines—the dual role

The actual cause of cell death induced in plants by oxidative stress still remains a conundrum. What remains a pertinent question is—whether PCD is induced by the ROS or is it the ROS levels itself which causes the cells to decease. Polyamine-derived H_2_O_2_ has been shown to participate in stress-induced cell wall stiffening and maturation (Angelini et al., 2010). In HIV-induced neuronal toxicity, Spm oxidase has been shown to act as a mediator of ROS production (Capone et al., 2013). Involvement of H_2_O_2_ in polyamine-induced cell death has been demonstrated in tobacco (*Nicotiana tabacum*) (Iannone et al., 2013) (Figure 6A). It is in this context that the level of H_2_O_2_ has drawn keen interest by raising doubts in the minds of researchers as to the way in which it leads to cellular damage inducing PCD, and the very fact that it may require a higher level of ROS to exterminate cells by direct oxidation. Studies applying oxidative stress to mutants deficient in different PCD pathways will be able to throw some light on this very question.

Many questions related to ROS metabolism have remained unanswered till date. We are thus trying to address these questions by considering the agency of molecules like H_2_O_2_ and polyamines both of which work toward a common target of stress tolerance. Intra-cellular levels of polyamines play a pivotal role by regulating a large number of processes through its metabolic pathways, including both anabolism and catabolism. Polyamine catabolism leads to an increase in level of H_2_O_2_, in turn influencing both stress damage and the response to stress damage (Pottosin and Shabala, 2014). Other by-products of the catabolic pathways include NO, GABA, acroliens, and others. GABA through TCA along with ornithine and other amino acids also acts as a signaling molecule. Studies demonstrate that external polyamine application not only alters polyamine homeostasis and metabolism systemically, but also affects the levels and the activity of antioxidative enzymes. Polyamines have been reported to be involved in the easing of oxidative stress through stimulation of two transcription factors SoxR and EmrR in *E. coli*. SoxR supposedly induces a superoxide response regulon transcription activator and *sodA* genes. These in turn influence the antioxidant machinery of the bacterial cells. Activation of EmrR and GshA together induces glutathione that has an inhibitory effect on H_2_O_2_ accumulation (Sakamoto et al., 2015) (Figure 6B). Scientists around the world look for such overlapping cues in plants, which help them to design their experiments and hypothesis.

A comprehensive analysis including biochemical, physiological, and molecular assays using microarrays and chips, coupled with proteomics will help us in deciphering the exact pathways both polyamines and H_2_O_2_ work on. Tanou et al. (2012, 2014) reported that a plant's response to salinity involves a crosstalk between polyamine transduction and oxidative/nitrosative signaling. Bruce et al. (2007) in their work have efficiently highlighted the benefits of long-term adaptation to biotic and abiotic stresses in plants and their evolutionary significance. They hypothesized that the plant retaliation toward any particular stress is regulated by a combination of their innate ability to combat stress as well as from previous exposure to a similar kind of stress. It was also opined that treating a plant with any kind of stress alleviator such as polyamines and in some cases even stress inducers such as H_2_O_2_ prior to the exposure to stress increases its chances of resistance and perennate (Shi et al., 2010). Li et al. (2015) analyzed the involvement of polyamines in the regulation of H_2_O_2_ and Ca^2+^ messenger systems associated with antioxidant defense and dehydrins in leaves of white clover resulting in water stress tolerance. However, further investigations are needed for establishing the phenomenon of polyamine-induced stress tolerance associated with H_2_O_2_ and Ca^2+^ signaling in different plant species and stress conditions.

The exposure to stress during very early development in plants is known as “priming.” There are reported examples of the effects of “priming” on physiological and biochemical responses that plants show on recurrent exposure to assortment variety of stresses (Jisha et al., 2013; Jisha and Puthur, 2016). Priming usually begins during seed germination, and might have long-lasting effects on the development of plants. This in fact may lead to adaptation to a diverse type of abiotic and biotic stresses. One can well imagine that it is priming that deals with the epigenetic changes, or markers, which indeed are responsible for carrying information over generations. Modifications of epigenetic regulations due to the changing environment on gene expression are extensively believed to be true; however, the appliance of such epigenetic adaptations is not well understood. The modifications generally occur at the chromatin level, and involve sequence-specific DNA methylation, histone acetylation, sumoylation and other similar abatements. While most of the epigenetic modifications are unwavering within the life of an organism, others are reversible, depending on growth and other regulations, and the rest appear to be transmitted to the subsequent generations through sexual reproduction (Sano, 2010; Shao et al., 2014; Sharma, 2014). From the various functions of polyamines and H_2_O_2_ studied till date, one can get a fair idea of the major roles played by them in priming for stress. Savvides et al. (2016) have argued about the role of some promising chemical agents such as sodium nitroprusside, H_2_O_2_, sodium hydrosulfide, melatonin, and polyamines that can potentially confer enhanced tolerance when plants are exposed to multiple abiotic stresses. In the present context it can be debated that increased polyamine accumulation in response to various stress conditions affects the epigenetic modifications of DNA and histones thus conferring stress tolerance. Plant polyamines create cellular responses during abiotic stress through modulation of ROS homeostasis via two distinct mechanisms (Takahashi and Kakehi, 2010). Firstly, polyamines promote ROS degradation by scavenging free radicals and activating antioxidant enzymes during stress conditions (Gupta et al., 2013a,b). Free polyamines are responsible for the detoxification of superoxide anions and H_2_O_2_, while the conjugated polyamines probably help in the scavenging of other ROS (Langebartels et al., 1991; Berberich et al., 2015). Kuznetsov and Shevyakova (2007) have reported that conjugated polyamines show more antioxidant ability than free polyamines. Secondly, polyamines promote ROS production through polyamine catabolism in the apoplast (Yoda et al., 2006; Mohapatra et al., 2010; Gupta et al., 2013a,b).

Mellidou et al. (2016) reported that a distinct crosstalk exists between peroxisomal polyamine oxidase and NADPH oxidase for maintaining ROS homeostasis in *A. thaliana* affecting their rate of respiration. They showed that the loss of function in AS-*SAMDC* salt-stressed plants resulted in an enormous increase of H_2_O_2_ compared to its control genotype. Moreover, higher SOD and elevated level of NADPH-oxidase activity in these SAMDC mutants emphasize the regulation of NADPH-oxidase derived H_2_O_2_ by SAMDC during salinity-induced stress in *Arabidopsis*. Work done by the same group also showed the NADPH-oxidase dependent stimulation of oxygen consumption in *Arabidopsis*. Peroxisomal AtPAO3 shows a decreased oxygen consumption rate in stark comparison to the loss-of-function in *Atpao3 Arabidopsis* plants, which show increased consumption through the AOX pathway (Andronis et al., 2014). Diphenyleneiodonium iodide (DPI) [but not ascorbate (ASA)] attenuates this increase, suggesting that NADPH-oxidase is upstream of a respiratory increase mediated by AOX. It is interesting to note that overexpressed *AtPAO3* plants show a balanced production of both O_2_^•−^ and H_2_O_2_, while *Atpao3* loss-of-function plants show a high O_2_^•−^/H_2_O_2_ ratio. Their data clearly suggests a well-defined cross-talk between NADPH-oxidase and AtPAO3 for balancing intracellular ROS affecting the cyt-c/AOX pathway. Lambeth and Neish (2014), while reviewing the relation of NOX (animal homolog of NADPH oxidase) and ROS, emphasized the role Nrf2/ARE signaling module in maintaining intracellular redox homeostasis in animals. However, till date no such pathway has been reported in plants. The activation of immune NADPH-oxidase by MAPK induced WRKY transcription factor throws some light on the transcriptional regulation of ROS in plants (Adachi et al., 2015).

**Figure 7:**
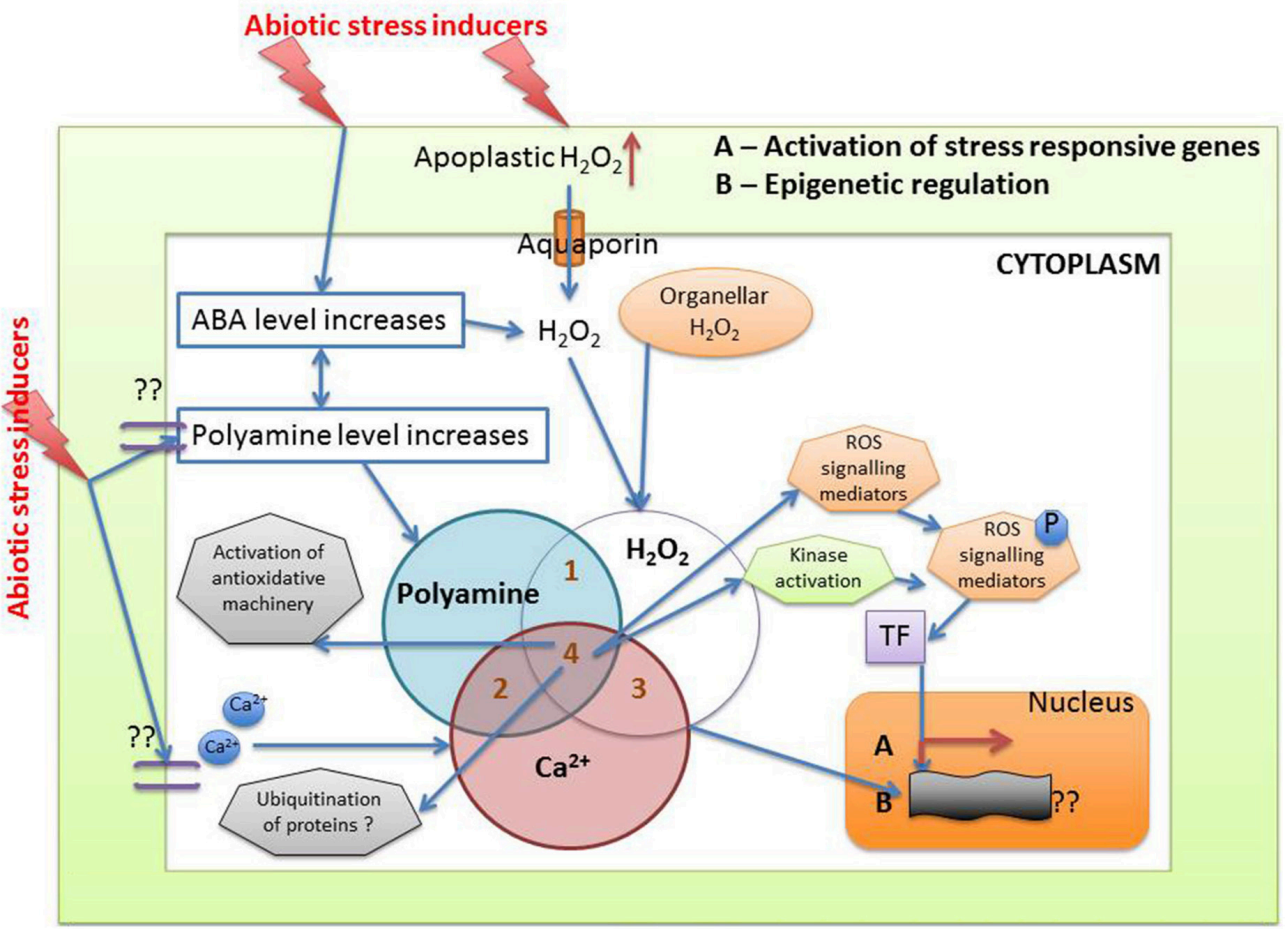
**Crosstalk between different metabolites during abiotic stress induced oxidative signaling (see text for details)**.

From the above discussion the following mechanism can be hypothesized: abiotic stress increases apoplastic and organellar H_2_O_2_ along with increased synthesis of higher polyamines and second messengers like Ca^2+^. Increased polyamine adds further to the H_2_O_2_ pool, which triggers the activation of antioxidative machinery (enzymatic and non-enzymatic) in the plants. ABA levels are also known to increase during stress, and this can be instrumental in triggering ROS mediated signaling pathways via polyamines. Epigenetic regulation of stress response and involvement of protein degradation also cannot be ruled out. While it is difficult to determine which of these mechanisms, during abiotic stress, is the most important one, it can be envisaged that a well-coordinated defense mechanism comprising of polyamines, Ca^2+^, H_2_O_2_ take part in response to oxidative stress in plants (Figure 7).

This review tries to present the dual role of both H_2_O_2_ and polyamines in a tabular form (Table 1). These evidences suggest that it is often “tricky” to establish a direct relationship between increased levels of polyamines/H_2_O_2_ and abiotic stress tolerance. Pál et al. (2015) have suggested that the statement “the higher polyamine level the better” cannot be generalized and elevated polyamine content might be the cause of stress-induced injury and/or of some protective mechanisms. We would like to suggest that the intracellular level of H_2_O_2_ and different forms of polyamines act as the “safety valve” of the whole plant system that is delicately balanced depending on the type and duration of the stress, developmental stage of the plant, the genotype it belongs to, and the type of plant tissue affected. Further studies will enable us to get a better understanding of the role of polyamine and H_2_O_2_ in plants using several modern era technologies. We earnestly hope that the dual role of these two key players playing a silent role in the backdrop will be unveiled in the near future.

**Table 1:** **Double role of H_**2**_O_**2**_ and polyamines in plant abiotic stress**.

**Metabolite**	**Role**	**Plant**	**Function**	**References**
H_2_O_2_	Positive	*Arabidopsis thaliana*	Increased expression of Nucleoside diphosphate kinase (NDP) leading to enhanced tolerance to several biotic and abiotic stresses	Moon et al., 2003
		*Vicia faba*	Intermediate in ABA signaling in guard cells	Pei et al., 2000
		*Arabidopsis thaliana*	Second messenger in ABA induced stomatal closure	Miao et al., 2006
		*Zea mays*	Induces salt tolerance by enhancing antioxidant metabolism and reducing lipid peroxidation in both leaves and roots	Azevedo-Neto et al., 2005
		Rice, Arabidopsis, Maize	Tolerance to biotic and abiotic stress by getting involved in various pathways	Reviewed by—Reczek and Chandel, 2015
		*Zea mays*	Pretreatment alleviates water loss during stress by increasing the level of soluble stress fighters like polyamine, sugars and proline	Terzi et al., 2014
	Negative	*Arabidopsis thaliana*	Indirectly activates WRKY53 transcription factor that leads to leaf senescence	Gadjev et al., 2008
		*Arabidopsis thaliana*	Influences Oxoglutarate-dependent dioxygenase gene in the cell death process	Gechev et al., 2005
		Ozone-fumigated Arabidopsis leaves	Apoplastic ROS accumulation as a result of activation of NADPH oxidases—leading to PCD	Joo et al., 2005
		*Nicotiana sylvestris*	Genetic alteration of the mitochondrial electron transport chain desensitizes the plant to stress-induced cell death	Dutilleul et al., 2003
		*Arabidopsis thaliana*	Enhanced ROS production such as H_2_O_2_ during drought induced senescence and heat stress	Lee et al., 2012, 2014
		*Nicotiana tabacum*	PCD is observed in plants deficient in the major catalase isoforms (ascorbate peroxidase and/or catalase)	Rizhsky et al., 2002
Polyamine	Positive	Sour orange plants (*Citrus aurantium*)	Influences oxidative and nitrosative status of plants exposed to salinity stress	Tanou et al., 2014
		*Oryza sativa*	Recovers salinity stress induced damage of plasma membrane (PM) and PM-bound H^+^- ATPase in salt-tolerant and salt sensitive rice cultivars	Roy et al., 2005
		*Arabidopsis thaliana* and *Nicotiana tabacum*	Transglutaminases catalyse the conjugation of polyamines to photosynthetic complexes and proteins and lead to enhanced photosynthetic activity under abiotic stress conditions	Hamdani et al., 2011; Ioannidis et al., 2012
		Different wheat cultivars	Different polyamines showed a variable increase during cold hardening	Szalai et al., 2009
		*Arabidopsis thaliana*	*Arabidopsis thaliana* plants overexpressing a *Cucurbita ficifoia* spermidine synthase gene have been demonstrated to become tolerant to multiple stress factors such as low temperature, freezing temperature, drought, salinity, and herbicide Paraquat	Kasukabe et al., 2004
		*Mesembrynthemum crystallinum*	Spermine inhibited the oxidative degradation of DNA by OH^−^	Kuznetsov and Shevyakova, 2007
	Negative	Tobacco plants	Transgenic tobacco plants overexpressing apoplastic PAO are not able to cope with oxidative burst generated by abiotic factors, causing detrimental effects	Moschou et al., 2008b; Moschou and Roubelakis-Angelakis, 2014
			The same plants mentioned above exhibited increased SOD and CAT expression, which do not exert a protective effect, but rather this increased expression represents an attempt to scavenge surplus H_2_O_2_ produced by continuous polyamine oxidation, which suggest that constitutive polyamine oxidation leads to chronic oxidative stress	Moschou et al., 2008b; Moschou and Roubelakis-Angelakis, 2014
		*Nicotiana tabacum*	Induction of hypersensitive cell death by H_2_O_2_ produced through polyamine degradation	Yoda et al., 2003
		Tobacco (*Nicotiana tabacum*) cultured cells	A gene encoding a tobacco *PAO* was isolated and used to construct RNAi transgenic cell lines. The results suggest that PAO is a key element for the oxidative burst, which is essential for induction of PCD, and that MAP kinase is one of the factors that mediate this pathway	Yoda et al., 2006
		*Arabidopsis thaliana*	Excess amount of exogenous thermospermine or spermine application resulted in an inhibition of leaf expansion, chlorophyll synthesis, and seed germination	Kakehi et al., 2008
		*Arabidopsis thaliana*	Exogenous thermospermine might also be oxidized, at least in part, by PAO and negatively affect the stem growth	Kakehi et al., 2008
		*Oryza sativa*	3-aminopropanal generated by polyamine back-conversion is a highly reactive aldehyde and is spontaneously deaminated to give acrolein. It is well known that in mammalian cell cultures, the toxicity of acrolein is higher than that of H_2_O_2_	Takano et al., 2012; Ono et al., 2012; Yoshida et al., 2009

## Conclusion

Environmental stresses are the key reasons behind massive crop loss throughout the world thus ravaging world agricultural economy. So the primary focus of the entire scientific world is to identify and implement strategies to overcome both abiotic and biotic stresses. In this process, they have identified roles of several molecules that are present within any normal living cell under non-stressful environmental conditions, playing the conventional roles that “Nature” designates them to play. But once the usual environment is replaced with a stressed one they start to function as stress alleviators. Two major examples of such molecules are polyamines and H_2_O_2_. Both of them have some demarcated responsibilities toward the cells they are produced in, but once they are exposed to an unfavorable environment they show a new dimension of their own which could not have been predicted earlier. Initially it was known that both polyamines and H_2_O_2_ play a significant role in maintaining physiological and biochemical processes in plants. However, their role in signaling and activating each other in response to stress is a new finding. Their correlation will be an intriguing topic for research in the near future. Some aspects of this interrelationship have already been discussed. But that is not enough to discern “whether polyamine and H_2_O_2_ functions as a dual-edged sword or not?” Despite the complexity plants are able to sort the stress signals and put up a well-organized defense mechanism. We would definitely be interested to know the intricate molecular mechanism that lies behind this unique inter-relationship and that is where the future prospect of this work lies.

## Author contributions

KG contributed in writing the manuscript and drawing the figures. AS and MC contributed in initial drafting of the manuscript, developing some of the figures, and have contributed equally. BG designed and developed the entire manuscript and contributed in writing and drawing the figures. KG and BG are the corresponding authors and also contributed in research fund management.

### Conflict of interest statement

The authors declare that the research was conducted in the absence of any commercial or financial relationships that could be construed as a potential conflict of interest.
